# Lipoic acid decreases breast cancer cell proliferation by inhibiting IGF-1R via furin downregulation

**DOI:** 10.1038/s41416-020-0729-6

**Published:** 2020-01-28

**Authors:** Diana Farhat, Sophie Léon, Sandra E. Ghayad, Nicolas Gadot, Philippe Icard, Muriel Le Romancer, Nader Hussein, Hubert Lincet

**Affiliations:** 1 Université Lyon 1, Lyon, France; 2 Inserm U1052, Centre de Recherche en Cancérologie de Lyon (CRCL), Lyon, France; 3 CNRS UMR5286, Centre de Recherche en Cancérologie de Lyon (CRCL), Lyon, France; 4 Lebanese University, Faculty of Sciences, Cancer biology Stem Cells and Molecular Immunology, Hadath-Beirut, Lebanon; 5 Plateforme Ex-Vivo, Département de Recherche Translationnelle et Innovation, SIRIC LYriCAN, INCa-DGOS-Inserm_12563, Centre Léon Bérard, Lyon, France; 6 Department of Biology, Faculty of Science II, Lebanese University, Fanar, Lebanon; 7 Plateforme Anatomopathologie-Recherche, Département de Recherche Translationnelle et Innovation, Centre Léon Bérard, Lyon, France; 8 Normandie Univ, UNICAEN, CHU de Caen Normandie, Unité de recherche BioTICLA INSERM U 119, 14000 Caen, France; 9 Service de chirurgie thoracique, Hôpital Cochin, Hôpitaux Universitaires Paris Centre, Paris, France; 10 ISPB, Faculté de Pharmacie, Lyon, France

**Keywords:** Cancer, Cell biology

## Abstract

**Background:**

Breast cancer is the second most common cancer in the world. Despite advances in therapies, the mechanisms of resistance remain the underlying cause of morbidity and mortality. Lipoic acid (LA) is an antioxidant and essential cofactor in oxidative metabolism. Its potential therapeutic effects have been well documented, but its mechanisms of action (MOA) are not fully understood.

**Methods:**

The aim of this study is to validate the inhibitory LA effect on the proliferation of various breast cancer cell lines and to investigate the MOA that may be involved in this process. We tested LA effects by ex vivo studies on fresh human mammary tumour samples.

**Results:**

We demonstrate that LA inhibits the proliferation and Akt and ERK signalling pathways of several breast cancer cells. While searching for upstream dysregulations, we discovered the loss of expression of IGF-1R upon exposure to LA. This decrease is due to the downregulation of the convertase, furin, which is implicated in the maturation of IGF-1R. Moreover, ex vivo studies on human tumour samples showed that LA significantly decreases the expression of the proliferation marker Ki67.

**Conclusion:**

LA exerts its anti-proliferative effect by inhibiting the maturation of IGF-1R via the downregulation of furin.

## Introduction

Lipoic acid (LA) is a natural compound synthesised de novo in small amounts by plants and animals. It is an essential coenzyme for mitochondrial enzymes, such as pyruvate dehydrogenase and α-ketoglutarate dehydrogenase.^1^ Aside from its important role in mitochondrial energy metabolism, it is also known for its powerful antioxidant effect.^2^ The benefits of LA treatment have been described in the management of several non-cancerous pathologies, including atherosclerosis, diabetes and neurodegenerative disorders.^3^ Beyond its efficacy in treating these chronic diseases, researchers have demonstrated its anti-proliferative effect on various cancers, including breast cancer.^4–8^ Breast cancer is the second most common cancer in the world, and by far, the most frequent cancer among women (25% of all cancers).

It is classified under several molecular subtypes, according to the presence or absence of oestrogen receptor α (ERα). This classification is important for disease prognosis, as well as for determining and providing an adequate therapy.

Despite advances in chemotherapy, hormonotherapy and targeted therapy in combination or not with surgery, the mechanisms of resistance remain the underlying cause of morbidity and mortality.^9^ In breast cancer, it has been demonstrated that the expression of the insulin-like growth factor-1 receptor (IGF-1R) increases in both ER-positive (ER+) and ER-negative (ER–) breast cancers and plays an oncogenic role.^10^ Moreover, many studies have shown the ability of IGF-1R to induce mammary tumour growth and metastases.^11–13^ Activation of IGF-1R in turn activates multiple signal transduction cascades, including PI3K/Akt and MAPK/ERK pathways,^14–16^ contributing to the promotion of breast cancer metastasis.

Indeed, both PI3K/Akt and MAPK/ERK pathways have been shown to be involved in various processes, such as cancer cell proliferation, migration and metabolism.^17^ Once Akt is activated, it stimulates the mammalian target of the rapamycin complex (mTORC1), which is a key regulator of cellular growth. In addition, the mTOR pathway is indirectly downregulated by activated AMPK, which is an energy sensor implicated in the regulation of protein and lipid metabolism in response to changes in fuel availability.^18^ Moreover, activation of AMPK is known to have anticancer effects in various cancer models, such as breast, colon and lung cancers.^19^ Activation of the pathways described above by IGF signalling requires the cleavage of pro-IGF-1R into its mature form. This maturation process is ensured by members of the pro-protein convertase family (PCs), including furin (PCSK3, PACE and SPC1). The latter is highly expressed in many types of cancers, including breast cancer, and was shown to promote the cancer cell proliferation, migration and invasion.^20^

In breast cancer, several studies demonstrated an anti-proliferative effect of LA.^4,5,21–25^ However, the precise molecular mechanisms by which LA elicits its anticancer properties remain largely unknown yet. In this study, we deciphered, for the first time, how LA inhibits the proliferation of breast cancer cells. As such, we investigated the inhibitory role of LA on the main proliferative pathways and on the maturation of their upstream effectors, notably IGF-1R. Our in vitro results demonstrate that LA inhibits IGF-1R maturation, and these results are supported by ex vivo findings.

## Materials and methods

### Lipoic acid

LA was purchased from Sigma (Sigma). A stock solution was prepared by dissolving LA in absolute ethanol and stocked at −20 °C. LA was diluted in culture medium for in vitro and ex vivo experiments.

### Cell lines, culture and siRNA transfections

ERα+ cells lines (MCF7, Cama1) and ERα– cell lines (MDA-MB-231, Skbr3 and HBL100) were obtained from the ATCC. All of the cell lines were maintained at 37 °C in the appropriate medium supplemented with 10% foetal bovine serum, 1% non-essential amino acid and 2% penicillin/streptomycin. All cell lines were regularly tested for mycoplasma contamination. We also used frozen tumours from human breast cancer-derived xenografts (PDX) from Dr. Marangoni. HBCc-12A- (ERα–) and HBCx-3-expressing (ERα+ ) cell lines were established from the human breast cancer xenografts as described by Marangoni et al.^26^

For knockdown experiments, *furin*-specific siRNAs from ThermoFisher Scientific (ambion s9988 and s9987) siRNA furin 1: sens: 5′-GGGCCUUCAUGACAACUCATT-3′; anti sens: UGAGUUGUCAUGAAGGCCCAG; siRNA furin 2: sens: GGUGGAAAAUGGACUGGCUTT; anti sens: AGCCAGUCCAUUUUCCACCTT; the scramble siRNA (Eurogentec) (20 nM) was transfected into MCF‐7 and MDA-MB-231 cells using the lipofectamine 2000 reagent according to the manufacturer’s instructions (Invitrogen).

### Cell proliferation assay by using IncuCyte technology

Cell proliferation was assayed using an IncuCyte® ZOOM Live-Cell Analysis System (Essen BiosScience, Sartorius), by collecting real-time data of cell confluence. Cells were seeded onto 96-well plates (5000 cells/well) and left to attach for 24 h. The cells were then treated with different concentrations of LA or absolute ethanol. Cell proliferation data were obtained by the cell confluence increment in each of the treatments, and expressed as percentage relative to that of control cells. Each sample was tested in triplicate.

### Western blot analysis

Cells were lysed using RIPA buffer (50 mM Tris HCl, pH 8, 150 mM NaCl, 1 mM EDTA, 1% NP-40 and 0.25% deoxycholate) supplemented with protease inhibitor tablets (Roche Molecular Biochemicals) and phosphatase inhibitors (1 mM NaF, 1 mM Na_3_VO_4_ and 1 mM β-glycerophosphate). Cell lysates were cleared by centrifugation (13,000 × *g*, 15 min, 4 °C), and protein concentration was determined by the Bradford method (Bio-Rad protein Assay). Proteins samples were separated on SDS-PAGE, and membranes were incubated with primary antibodies overnight at 4 °C on a shaker with the following primary antibodies: anti-IGF-1R (Cell Signaling), anti-IR-α (Cell Signaling), anti-EGFR (Cell Signaling), anti-furin (Proteintech), anti-Akt and anti-pAkt (Cell Signaling), anti-ERK and anti-pERK (Cell Signaling), anti-S6 and anti-pS6 (Cell Signaling), anti-S6K1 and anti-pS6K1 (Cell Signaling), anti-AMPK and anti-pAMPK (Cell Signaling) and anti-Tubulin (Sigma). Membranes were washed with TBS-Tween, and incubated with anti-rabbit or anti-mouse secondary antibodies. Detection was carried out using ECL Prime reagent (GE Healthcare).

### RNA isolation, reverse transcription and quantitative real-time polymerase chain reaction (RT-qPCR)

The total RNA was isolated using TRIzol (Invitrogen) according to the manufacturer’s instructions. RNA was reverse transcribed to complementary DNA (cDNA) using SuperScript™ II Reverse Transcriptase (Life Technologies). The primers are listed as follows: *IGF-1R* forward: 5′-TGTCCAGGCCAAAACAGGA-3′ and reverse: 5′-CGGGTTCACAGAGGCATACA-3′; *furin* forward: 5′-TATGGCTACGGGCTTTTGG-3′ and reverse: 5′-TTCGCTGGTGTTTTCAATCTCT-3′; 28S forward: 5′-CGATCCATCATCCGCAATG-3′ and reverse: 5′-AGCCAAGCTCAGCGCAAC-3′. RT-qPCR was performed using the Bio-Rad CFX 96 Real-time PCR system (Bio-Rad) using SsoAdvanced™ Universal SYBR® Green Supermix according to the manufacturer’s instructions. The mRNA level was normalised to 28S using Livak’s method (2^−(ΔΔCq)^) method. RT-qPCR conditions were as follows: 1 activation step for 5 min at 94 °C, 35 cycles of denaturing at 94 °C for 15 s, primer annealing at 59 °C for 45 s and primer extension at 72 °C for 2 min and a final extension at 72 °C for 5 min.

### Immunofluorescence analysis

In total, 80,000 MCF7 cells were seeded onto glass coverslips and incubated with LA for several time points (24 and 48 h). The cells were fixed in methanol for 2 min and washed once with cold PBS. We used rabbit anti-IGF-1R as a primary antibody (Cell Signaling), and fluorescein isothiocyanate (FITC)-conjugated goat anti-rabbit IgG (Jackson ImmunoResearch) as a secondary antibody.

### Ex vivo assays

Fresh human mammary samples were obtained from chemotherapy-naive patients with invasive carcinoma after surgical resection at the Centre Léon Bérard (CLB, Lyon, France). As required by the French Committee for the Protection of Persons, informed consent was obtained from patients to use their surgical specimens and clinicopathological data for research purposes, and the local ethics committee approved the protocols. Nine tumours were cut into thin slices of 250 µm using a vibratome (HM 650 V Microm) and incubated for 48 h with or without 2 mM of LA (Table 1). Slices were then fixed in 4% paraformaldehyde and paraffin embedded. Sections (4 µm) were then cut for standard histological analysis assessed by haematoxylin phloxin saffron (HPS) staining and immunochemistry analysis using the Ki67 antibody (Cell Signaling), which is a marker of proliferation. The percentage of positive cells among 200 cancer cells was counted.

**Table 1:** Main clinical characteristics of nine human breast cancer samples used for ex vivo assay.

Tumour	Grade	ER α status	PR status
1	II	+	−
2	I	+	+
3	II	+	+
4	I	+	+
5	III	−	−
6	III	−	−
7	II	+	+
8	III	−	−
9	I	+	+

### Immunohistochemistry analysis

Immunohistochemistry was performed on 4-mm-thick sections of formalin-fixed, paraffin-embedded and heat-treated (for antigen retrieval) tissues (DakoCytomation). Sections were stained with haematoxylin–eosin–safran, and for immunohistochemistry detection, the primary antibodies used were anti-IGF-1R (Cell signaling) and anti-Ki67 (Cell Signaling). Diaminobenzidine was used as chromogen. Images were acquired using a Zeiss Axiovert. The whole slide was scanned automatically with the Histolab 6.2.0 MICROVISION Instrument system.

### Statistical analysis

Data are presented as mean ± SEM of three independent experiments. Unpaired two-tailed Student’s *t* test was used for statistical analysis. A *P* value of < 0.05 was considered statistically significant. *P < 0.05; **P < 0.01; ***P < 0.001. The graph artwork was created using Microsoft office Excel 2010 and GraphPad prism 6.

## Results

### LA inhibits the proliferation of breast cancer cells in an ERα-independent manner

**Fig. 1:**
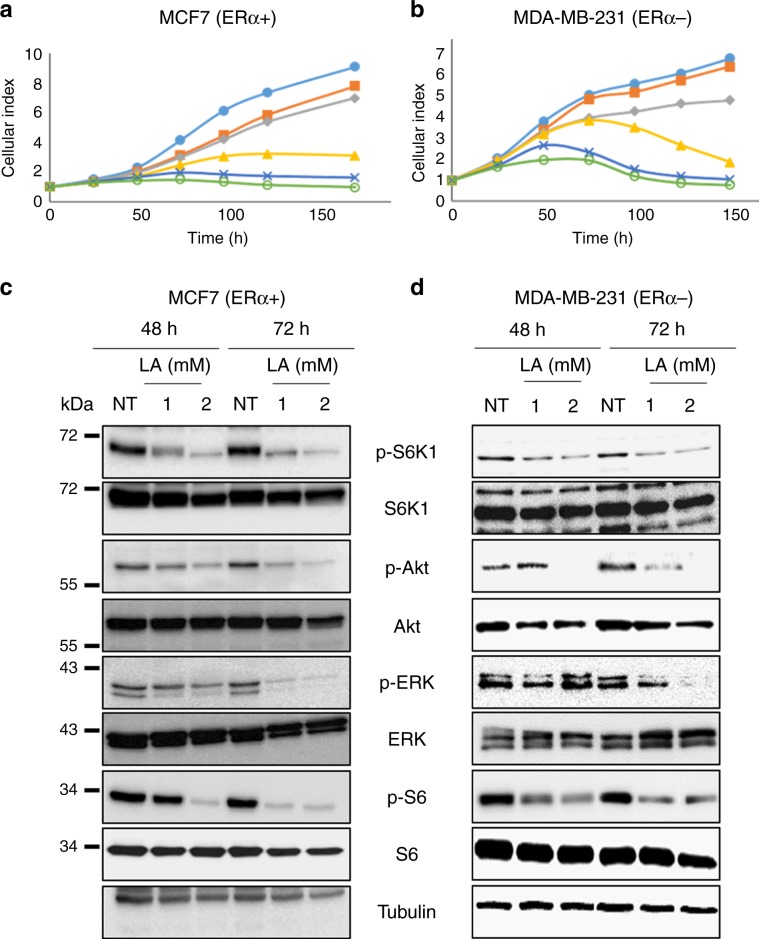
Dose-dependent effect of lipoic acid on the proliferation of ERα + and ERα− breast cancer cell lines. ERα+ cell line MCF7 and ERα− cell line MDA-MB-231 were treated with LA at increasing concentrations (0.5, 1, 2 and 5 mM) followed by assessment of cell growth using the IncuCyte ZOOM technology. **a** MCF7 cell line. **b** MDA-MB-231 cell line. These experiments were performed in triplicate, and repeated three independent times with similar results. Western blot analyses of LA (1 or 2 mM) reducing PI3K/Akt and ERK downstream effectors in **c** MCF7 (ERα+) and **d** MDA-MB-231 (ERα−) cell lines.

To investigate the effect of LA on breast cancer cell proliferation, we treated the cells with MCF7, and MDA-MB-231 breast cancer cell lines were treated with different concentrations of LA and monitored their viability using the IncuCyte real-time imaging system. We first studied its role on ERα+ cell lines (MCF7, Cama1 and HBCx-3). LA treatment at 0.5 mM was able to reduce the proliferation of Cama1 cell line. However, this concentration of LA had little effect on MCF7 and HBCx-3 cell lines, while increasing concentrations totally inhibited cell proliferation (Fig. 1a; Supplementary Data, Fig. 1). The same results were obtained for ERα− cell lines (MDA-MB-231, Skbr3 and HBCc-12A) (Fig. 1b; Supplementary Data, Fig. 1). In contrast, LA did not inhibit the proliferation of non-malignant HBL100 cells (Supplementary Data, Fig. 1). These results demonstrate that LA inhibits the proliferation of cancer cells independently of their ERα status, but not that of non-tumorigenic cells. Owing to the similarities in the results obtained between cell lines, our subsequent experiments focused on an ERα+ and an ERα− cell line, namely MCF7 and MDA-MB-231, respectively.

### LA inhibits ERK and Akt pathways and activates the AMPK pathway in breast cancer cell lines in an ERα-independent manner

It is well known that ERK and Akt pathways play a central role in cell proliferation.^27^ We thus investigated the effect of LA on these two pathways. As shown in Fig. 1c, in MCF7 cells, LA strongly reduced ERK phosphorylation at 1 mM and 2 mM at 48 h, while at 72 h, ERK phosphorylation was totally abolished as compared with control cells treated with 2% of EtOH (NT). In MDA-MB-231 cells, LA blocked ERK phosphorylation only after 72 h of treatment and at 2 mM (Fig. 1d). In addition, LA prevented Akt phosphorylation on Ser 473 in MCF7 cells at all treatment time periods and concentrations (Fig. 1c). In MDA-MB-231 cells, 2 mM of LA also blocked Akt phosphorylation on Ser 473 from 48 h onwards (Fig. 1d). As depicted in Fig. 1c, d, LA treatment totally inhibited Akt-induced S6K phosphorylation, which is present downstream of the Akt signalling cascade. Moreover, LA partially inhibited p-ribosomal protein S6 (S6) phosphorylation, which is downstream of S6K in both cell lines.

We also evaluated whether LA activates AMPK signalling by phosphorylation of AMPK on Thr 172, because activated AMPK specifically downregulates the mTOR signalling pathway, leading to the inhibition of cell proliferation. The level of AMPK phosphorylation increased in a time- and concentration-dependent manner in MCF7 cells (Supplementary Data, Fig. 2A). In MDA-MB-231 cells, LA increased AMPK phosphorylation only at 2 mM of LA at 48 h and 72 h (Supplementary Data, Fig. 2B). These results show that LA inhibits proliferation of breast cancer cells by simultaneously targeting ERK and Akt pathways. Moreover, these results show AMPK, which activates the tuberous sclerosis (TSC) 1/2 complex, which is a negative regulator of mTORC1.^28^

### LA suppresses IGF-1R expression at the protein level in breast cancer cells

**Fig. 2:**
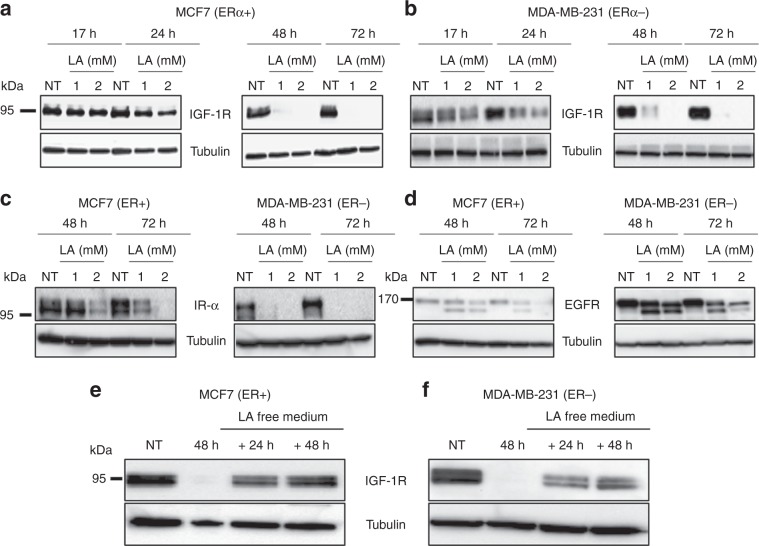
LA reduces the expression of several TKRs in MCF7 (ERα + ) and MDA-MB-231 (ERα−) cell lines via a reversible mechanism. MCF7 and MDA-MB-231 breast cancer cell lines were treated with EtOH 2% (NT) or with LA (1 or 2 mM) for 48 and 72 h. The effect of LA (1 or 2 mM) on IGF-1R level after 17, 24, 48 and 72 h of treatment was evaluated by western blotting in **a** MCF7 (ERα+ ) and **b** MDA-MB-231 (ERα−) cell lines. The effect of LA (1 or 2 mM) on **c** IR-α level and **d** EGFR level after 48 and 72 h of treatment was detected by western blotting in MCF7 (ERα+) and MDA-MB-231 (ERα−) cell lines. LA has a reversible effect on IGF-1R downregulation. IGF-1R is re-expressed after 24 and 48 h in lipoic acid-free medium. IGF-1R protein level was detected by western blot analysis in **e** MCF7 (ERα+) and **f** MDA-MB-231 (ERα−) cell lines treated with LA (2 mM) for 48 h followed by 24 or 48 h of incubation with LA-free media.

In order to decipher how LA inhibits the PI3K/Akt and MAPK/ERK signalling pathways, as these pathways are major downstream pathways of several tyrosine kinase receptors involved in cell proliferation and survival, we studied the expression of IGF-1R, IR and EGFR upon LA treatment. As shown in Fig. 2a, LA treatment of MCF7 cells decreased the IGF-1R expression level in a time- and concentration-dependent manner. From 24 h onwards, at 2 mM of LA, we observed a slight decrease in the level of IGF-1R expression. Of note, at 72 h, no band was detected for the IGF-1R protein, irrespective of the LA concentration used (1 or 2 mM). In MDA-MB-231 cells, 2 mM LA treatment resulted in a slight decrease in the level of the IGF-1R protein from 17 h onwards (Fig. 2b). Forty-eight hours after LA treatment (1 or 2 mM), IGF-1R expression was almost lost. The effect of LA on the reduction of IGF-1R expression level was faster in the MCF7 cell line than in MDA-MB-231 cells. IGF-1R downregulation under LA treatment was also detected in other breast cancer cell lines (Supplementary Data, Fig. 3). Interestingly, we found that LA also reduces the expression of other tyrosine kinase receptors, the IR and EGFR in a concentration- and time-dependent manner in both cell lines (Fig. 2c, d).

Next, we wondered whether LA could impact IGF-1R transcription, by conducting RT-qPCR experiments. No decrease in *IGF-1R* mRNA was observed, irrespective of LA concentration and exposure (Supplementary Data, Fig. 4). These data showed that this inhibitory effect did not occur at the transcriptional level, but rather at the translational or post-translational levels.

Our results showed that LA at 1 or 2 mM represses IGF-1R expression 48 h after exposure. We then evaluated whether this effect was reversible or not. As such, cells were treated with 2 mM of LA for 48 h and then incubated in the LA-free medium for 24 or 48 h. In the two cell lines, we observed the reappearance of IGF-1R expression 24 h following LA withdrawal, and a continuous increase until 48 h (Fig. 2e, f). Hence, these results reveal that LA induces a transient decrease in IGF-1R protein expression.

### LA promotes the accumulation of pro-IGF-1R and reduces IGF-1R plasma membrane localisation

**Fig. 3:**
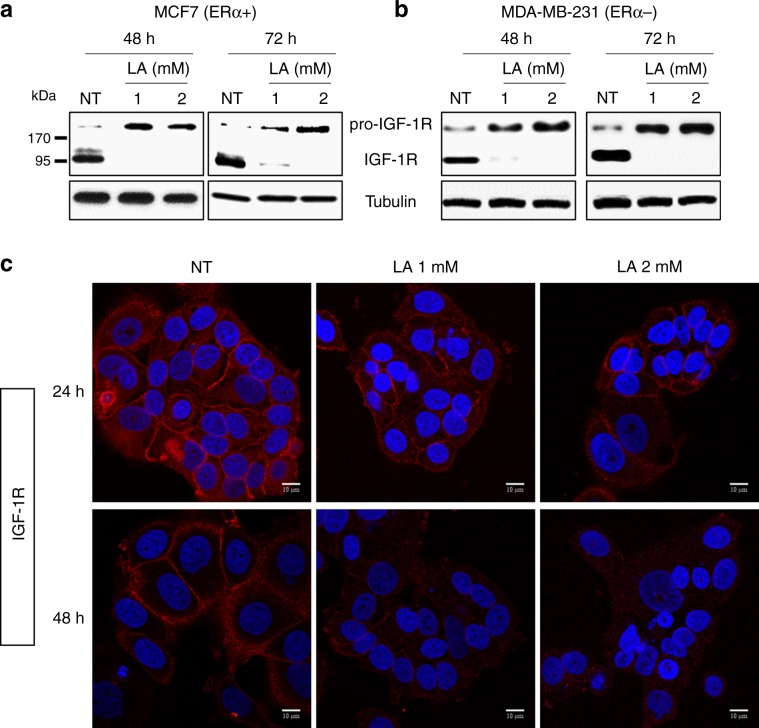
LA promotes pro-IGF-1R accumulation and reduces mature IGF-1R expression. MCF7 and MDA-MB-231 breast cancer cell lines were treated with EtOH 2% (NT) or with LA (1 or 2 mM) for 24, 48 and 72 h. Pro-IGF-1R and mature IGF-1R expressions were detected by western blot analysis after 48 and 72 h of LA treatment (1 or 2 mM) in **a** MCF7 (ERα+) and **b** MDA-MB-231 (ERα−) cell lines. **c** IGF-1R protein expression and localisation were evaluated by immunofluorescence in MCF7 cells treated with 1 or 2 mM of LA for 24 and 48 h, as indicated.

Based on our previous results, we hypothesised that LA could have an effect on the post-translational processing of pro-IGF-1R into mature and active IGF-1R. Indeed, we observed an accumulation of the pro-IGF-1R in response to LA treatment from 48 h onwards in MCF7 and MDA-MB-231 cells, while mature IGF-1R was totally absent (Fig. 3a, b). Similarly, the pro-IR form increased in these cell lines in response to LA treatment, whereas we observed a reduction in the mature IR form (Supplementary Data, Fig. 5A, B). It is well known that IGF-1R is localised at the level of the plasma membrane, whereas pro-IGF-1R has a cytoplasmic localisation.^29^ We then tested the localisation of IGF-1R in the MCF7 cell line upon LA treatment by immunofluorescence confocal microscopy. Twenty-four hours after LA treatment, a partial loss of IGF-1R at the plasma membrane was detected in favour of the cytoplasmic localisation, compared with control cells (Fig. 3c). In coherence with these results, we have demonstrated by western blot that LA (1 or 2 mM) treatment for only 24 h is not sufficient to abolish the total expression of processed IGF-1R and to induce a total shift to its uncleaved form (data not shown). Thus, these results explain why there is still detectable IGF-1R at the plasma membrane after LA (1 or 2 mM) treatment for 24 h (Fig. 3c).

This loss of IGF-1R at the plasma membrane increased until 48 h, and no plasma membrane expression was observed at this time (Fig. 3c).

### LA inhibits the maturation of pro-IGF-1R by targeting the pro-protein convertase furin

**Fig. 4:**
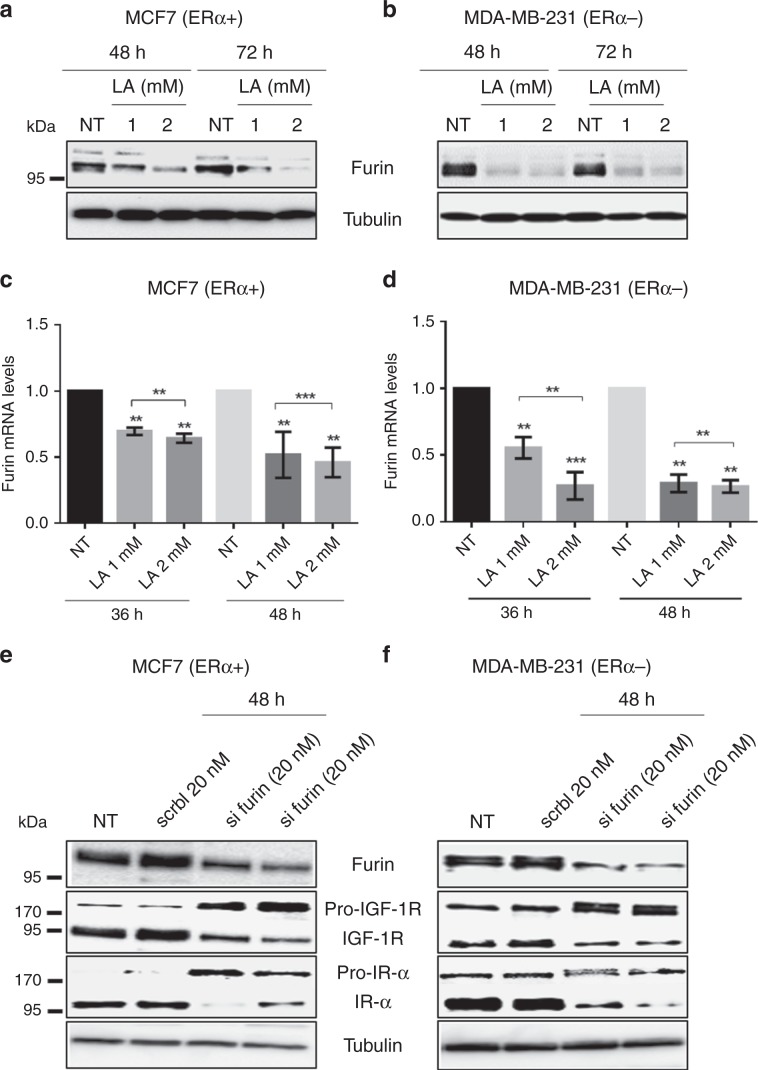
LA reduces furin mRNA and protein levels in breast cancer cell lines. Furin silencing inhibits the maturation of IGF-1R and IR-α, or furin silencing promotes the pro-IGF-1R and pro-IR accumulation and decreases the IGF-1R and IR-α expression in breast cancer cell lines. MCF7 and MDA-MB-231 breast cancer cell lines were treated with EtOH 2% (NT) or with LA (1 or 2 mM) for 36, 48 and 72 h. The furin protein expression was detected by western blot analysis after 48 and 72 h of LA treatment (1 or 2 mM) in **a** MCF7 (ERα+) and **b** MDA-MB-231 (ERα−) cell lines. The furin mRNA level was assessed by real-time quantitative reverse transcription PCR in **c** MCF7 (ERα+) and **d** MDA-MB-231 (ERα−) cell lines treated with LA (1 or 2 mM). Data are normalised against 28S mRNA level used as an endogenous control. The results are expressed relative to the levels in control cells set at one. This analysis was performed three times. Pro-IGF-1R, pro-IR, mature IGF-1R and mature IR-α expressions were detected by western blot analysis in **e** MCF7 (ERα+) and **f** MDA-MB-231 (ERα−) transfected with two different furin siRNAs or scramble siRNA for 48 h.

The pro-protein convertase, furin, catalyses the endoproteolytic cleavage of the inactive pro-IGF-1R into its active form.^30,31^ Thus, we investigated whether LA treatment had an effect on its expression. Forty-eight hours after LA treatment, the expression of furin decreased in MCF7 and MDA-MB-231 cell lines (Fig. 4a, b), in a time- and concentration-dependent manner. Indeed, 72 h after incubation in 2 mM of LA, the expression of furin decreased notably in the MCF7 cell line compared with MDA-MB-231 cells (Fig. 4a, b). We confirmed that LA downregulates the furin protein expression by validating the loss of maturation of other furin substrates such as Notch 3 (Supplementary Data, Fig. 6).

We also examined the mRNA level of *furin* by RT-qPCR to determine whether this downregulation is due to an inhibition of the transcriptional activity in response to LA. Cell lines were treated with 1 or 2 mM of LA for 36 or 48 h. A significant reduction in *furin* mRNA was observed after treatment with LA compared with untreated MCF7 or MDA-MB-231 cells (Fig. 4c, d). Next, we evaluated whether this effect was reversible. To achieve this, cells were treated with 2 mM of LA for 36 or 48 h, and then incubated in LA-free medium for 24 h. As described in the Supplementary Data, Fig. 7A, B, the level of *furin* mRNA increased in MCF7 and MDA-MB-231 cell lines following the removal of LA. Thus, our results show that LA acts on the maturation process of pro-IGF-1R and pro-Notch into active forms by reversibly inhibiting *furin* expression. In order to prove the implication of furin in IGF-1R and IR maturation in our cellular models, we transfected ERα+ and ERα− breast cancer cell lines with furin or scramble siRNA for 48 h. We demonstrated that *furin* silencing leads to an accumulation of their pro-forms (Fig. 4e, f). Indeed, our results showed that siRNA-mediated silencing of *furin* had a profound effect on the processing and maturation of IGF-1R and IR.

### Preclinical evaluation of exposure to LA on human mammary tumours

**Fig. 5:**
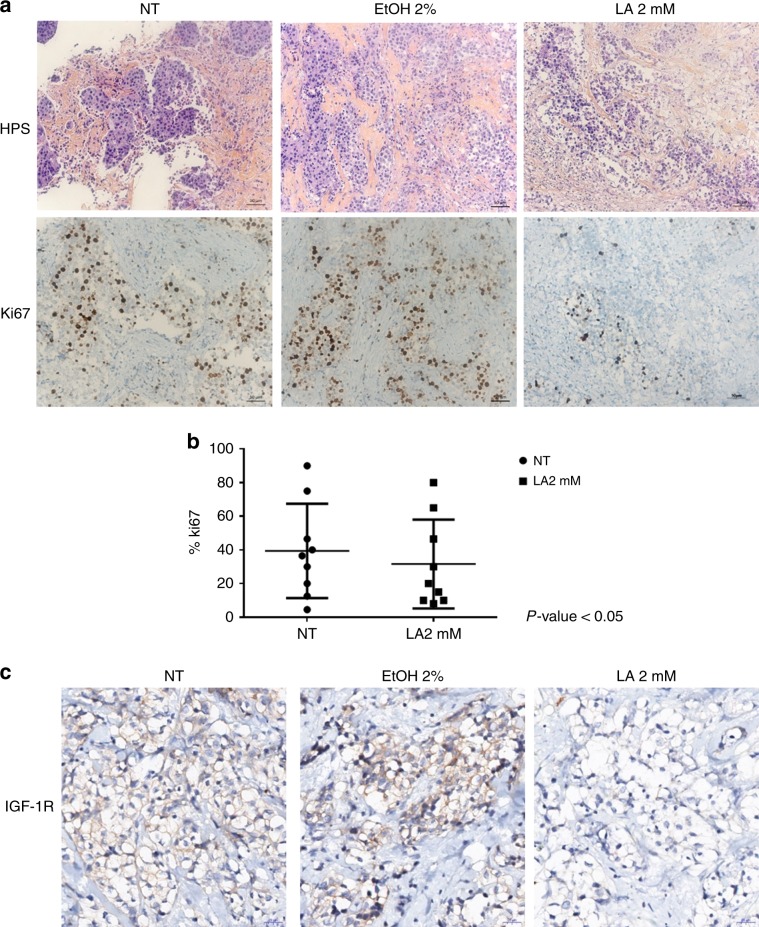
LA decreases the expression of Ki67 and IGF-1R. IHC was conducted on nine breast tumours. Paraffin sections were stained with standard haematoxylin–eosin–safran stains. **a** Ki67 labelling was performed 48 h after LA (2 mM) or 2% ethanol treatment in addition to the non-treated tumours. Nuclear localisation was noted (thick black arrows). Harris’ haematoxylin counterstain. **b** Scatterplot showing the Ki67 staining mean score in LA-treated tumours compared with non-treated (NT) tumours; unpaired two-tailed Student’s *t* test was used for statistical analysis with *P* value < 0.05. **c** IGF-1R labelling was done 48 h after LA (2 mM) or 2% ethanol treatment in addition to the NT tumours.

Next, we tested the preclinical effects of LA on fresh human mammary tumour samples after surgical resection. In effect, we evaluated the response of nine breast tumours to LA using an ex vivo organotypic culture assay, in which the tumour microenvironment is closely mimicked to evaluate its clinical potential. The tumours from patients with untreated breast cancer were included, five of which were positive for ERα and four negative for this receptor. IHC scores of positive cells for the proliferation marker Ki67 and the HPS staining were determined in the epithelial tumour cell compartments. Figure 5a illustrates their IHC detection on a representative tumour sample treated or not with 2 mM of LA for 48 h. We observed a decrease in Ki67 in response to LA compared with the non-treated condition. Among the nine tumours, Ki67 staining displayed a mean score of 30% (ranging from 10 to 98%) in LA-treated tumours compared with 43% (ranging from 5 to 80%) in non-treated tumours (Fig. 5b). Moreover, we detected the IGF-1R expression by IHC on these tumour samples. Only three out of nine tumour samples expressed IGF-1R at the basal level. In these three cases, our results showed a repression of IGF-1R in response to LA compared with non-treated tumours or 2% absolute ethanol condition (Fig. 5c). IGF-1R downregulation obtained by the ex vivo approach corroborates with our in vitro results.

## Discussion

LA, an essential cofactor of a mitochondrial respiratory enzyme, exhibits antitumour activities in several cancer models such as thyroid, bladder, ovarian, colon, lung and breast cancer by acting on most of the signalling pathways implicated in proliferation, invasion, migration, EMT, stemness and apoptosis.^1,6,8,21,32–37^ Several studies demonstrated the beneficial effects of LA on some breast cancer cell lines by reducing the breast cancer cell viability, proliferation and metastasis process, and by promoting apoptosis.^4,5,21–23^ However, the mechanisms by which LA elicits its anticancer effects remain unclear. Herein, we demonstrate the inhibitory effect of LA on the proliferation of a large number of ERα+ and ERα− breast cancer cell lines. Regardless of the ERα status, LA was able to inhibit crucial signalling pathways such as PI3K/Akt/mTOR and ERK in a time- and concentration-dependent manner. It is known that these pathways are directly implicated in cancer progression and especially cancer cell proliferation.^38,39^ Under our experimental conditions, we demonstrated that LA inhibits these pathways by suppressing the phosphorylation of Akt and ERK. Moreover, LA reinforces the Akt inhibition by activating AMPK through its phosphorylation. Accordingly, once AMPK is activated, it phosphorylates and thereby activates the TSC complex leading to mTOR inhibition.^40^ Furthermore, we also showed that LA inhibits the downstream effectors of mTOR, S6K1 and S6, by reducing their phosphorylation in a time- and concentration-dependent manner. The latter were previously reported to be involved in the translation process in cancer cells.^41^ In line with these studies, our results suggest that LA could reduce the translation of proteins required for cancer cell growth and survival, such as cyclin, c-Myc and bcl-2.^42,43^

To gain further insight into these mechanisms, we studied the expression of the tyrosine kinase receptor, IGF-1R, which is an attractive therapeutic target in breast cancer, in response to LA treatment. Of note, the correlation between IGF-1R overexpression and cancer development, as well as the role of its downstream signalling cascade, such as activation of the PI3K/Akt pathway, in the promotion of proliferation of breast cancer cell lines, have been reported in several studies.^44–46^ Dunn et al.^47^ also demonstrated that suppression of IGF-1R expression leads to inhibition of metastasis, invasion and adhesion of breast cancer cells.^47^ In this context, targeting IGF-1R appears to be a promising approach for inhibiting tumour growth and increasing therapeutic efficacy. Interestingly, our results showed that LA reduces IGF-1R expression from 24 h onwards, and that this expression was abolished at 48 h upon treatment with 1 mM LA in several ERα+ and ERα− cell lines. Moreover, we demonstrated that this downregulation did not occur at the transcriptional level but rather at the post-translational level, since this dramatic decrease in IGF-1R was coupled with an accumulation of the immature (pro-IGF-1R) form upon LA treatment in a time- and concentration-dependent manner. These results were confirmed by immunofluorescence, highlighting a change in the localisation of IGF-1R from the plasma membrane to the cytoplasm. Herein, the cytoplasm localisation of IGF-1R is an indication of its deactivation.^30,48^ However, it is not always the case. In this context, it has long been recognised that ligand binding at the plasma membrane induces internalisation of activated IGF-1R.^49^

Furthermore, based on previous studies,^30,31^ IGF-1R maturation is processed by the pro-protein convertase furin. This enzyme is enriched in the Golgi, and this is where cleavage of IGF-1R pro-receptor into an active form is thought to occur. Moreover, furin is also involved in processing of IGF 1/2.^50^ It is well known that furin is overexpressed in cancer, and it is considered as a tumour progression marker or as a prognostic factor.^51^ For this reason, researchers were interested in determining its role in cancer progression and promotion. Accordingly, several studies have considered furin as an effective anticancer target.^51–53^ Indeed, we herein demonstrated that LA reduces *furin* mRNA levels, leading to a decrease in its protein expression.

In addition, IGF-1R is highly homologous to another tyrosine kinase receptor, namely the insulin receptor (IR).^54^ Of note, numerous in vitro and in vivo studies have demonstrated the correlation between IR overexpression and malignancy in several cancer models, including breast cancer.^55^ The two structurally different isoforms of IR, IR-α and IR-β, are overexpressed in many cancers.^56^ IR-α is the main isoform that plays an important role in cancer cell stemness, in tumour progression and in resistance to IGF-1R-targeted therapies (for review, see ref. ^56^). For these reasons, we also studied the expression of IR upon LA treatment. Interestingly, our results showed that IR-α is abolished in favour of the immature form (pro-IR) upon LA treatment (1 and 2 mM) in our breast cancer cell lines. Collectively, our results show that LA inhibits the IGF-1R and IR-α maturation by reducing furin expression. This work reveals the important role of LA in cancer treatment, thanks to its dual inhibition of the IGF-1R/IR network, which is a serious obstacle to treatment efficacy.^52^

Moreover, our results have shown that LA reduces the EGFR expression with appearance of a doublet by western blots (Fig. 3d). Of note, the EGFR ligands are expressed as transmembrane precursors. Their activation is realised by their cleavage by ADAM17, which is a furin substrate. After cleavage, these ligands can bind and activate EGFR.^57^ Based on these data and our results, we can hypothesise that the downregulation of furin upon LA treatment could induce the accumulation of the pro-ADAM17 (inactive form), which then could inhibit the release of EGFR ligands and their binding on their receptor. These events could induce EGFR internalisation and degradation.

The ex vivo model presented herein enabled us to replicate the microenvironment of intact breast tumour tissues. This approach maintains specific interactions between tumour cells and the surrounding normal tissue components, and supplies a powerful and reproducible tool to study the response of fresh individual tumours to anticancer drugs. We tested a series of nine human primary breast tumours in this ex vivo assay. Our results showed that five tumours revealed a significant decrease in Ki67 levels in response to LA. Moreover, among these nine tumours, only three tumours expressed IGF-1R at the basal level, and this expression decreased upon LA treatment. Thus, these results confirm the beneficial effects of LA on breast cancer cells in their microenvironment.

**Fig. 6:**
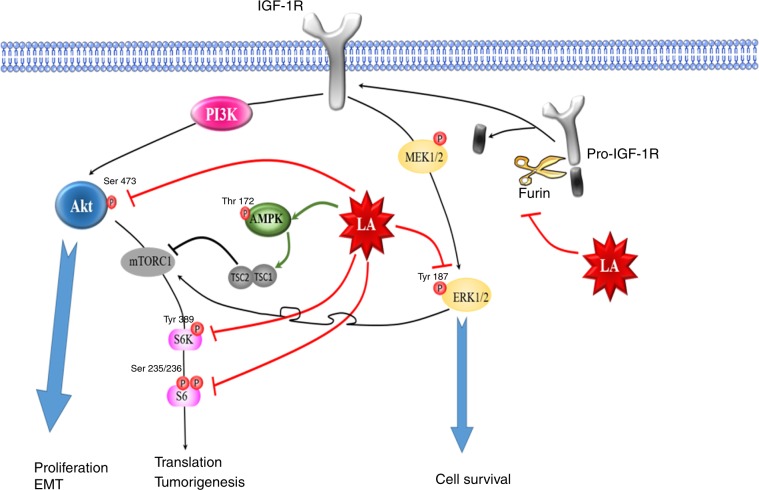
Schematic representation of the anti-proliferative role or anticancer effects of lipoic acid in breast cancer cells. Cellular proliferation, survival and EMT are controlled by the activation of ERK and PI3K/Akt pathways. LA prevents the phosphorylation of ERK, in particular by blocking the activation of c-Myc, which is a centrepiece of the several processes implicated in cancer development. Moreover, LA inhibits the activation of Akt, leading to the inhibition of mTOR effectors S6K1 and S6, which induce the translational process; thus, LA reduces cellular proliferation and growth processes. On the other hand, LA activates the AMPK protein that negatively regulates the Akt pathway, thereby reducing the translation of several proteins involved in the tumorigenesis process. Moreover, LA impedes the activation of ERK and PI3K/Akt pathways by the inhibition of some TKR expression, such as IGF-1R, which is an upstream activator of these pathways, reinforcing its inhibition. IGF-1R maturation is inhibited by LA by downregulating the furin expression, leading to a decrease of mature IGF-1R at the membrane level.

Taken together, we herein show that LA inhibits the proliferation of various ERα+ and ERα− breast cancer cell lines in vitro and ex vivo by inhibiting the maturation of IGF-1R (Fig. 6). This inhibition is the consequence of the reduction of furin expression, which is implicated in tumour aggressiveness. Nevertheless, the mechanism underlying LA downregulation of furin expression remains unclear. Though LA could be considered as a potential agent for the treatment of cancer, possibly as a supplement to systemic anticancer treatments, further in vivo studies are needed to evaluate its efficacy in combination with conventional therapy.

## Supplementary information

supplementary data

## Data Availability

All data presented within the article and its supplementary information files are available upon request from the corresponding author.
