# Supplementary material for: Lipoic acid decreases breast cancer cell proliferation by inhibiting IGF-1R via furin downregulation
Source: Br J Cancer. 2020 Jan 28;122(6):885–94. doi: 10.1038/s41416-020-0729-6 (PMC7078196; doi:10.1038/s41416-020-0729-6)

#### **Supplementary Data**

Figure 1: Dose-dependent effect of lipoic acid on the proliferation of ERα+ and ERα- breast cancer cell lines. ERα+ cell lines (Cama1 and HBCx-3), ERα- cell lines (Skbr3 and HBCc-12A) and nonmalignant cell line (HBL100) were treated with LA at increasing concentrations (0.5, 1, 2 and 5 mM) followed by assessment of cell growth using the IncuCyte ZOOM technology. **A:** ERα+ cell lines; **B:** ERα- cell lines. LA did not reveal a cytotoxic effect in **C**: nonmalignant cell line treated with increasing concentrations of LA. These experiments were performed in triplicate and repeated three independent times with similar results.

Figure 2: Lipoïc acid induces AMPK phosphorylation in **A:** MCF7 and **B**: MDA-MB-231 cell lines 48 hrs and 72 hrs after treatment.

Figure 3: LA inhibits the expression of IGF-1R in several ERα+ and ERα- breast cancer cell lines. These cell lines were treated with EtOH 2 % (NT) or with LA (1 or 2 mM). Effect of LA (1 or 2 mM) on IGF-1R level after 48 and 72 hrs of treatment was evaluated by western blot analysis in **A**: ERα+ cell lines (Cama1 and HBCx-3); **B**: ERα- cell lines (Skbr3 and HBCc-12A).

Figure 4: LA does not regulate IGF-1R mRNA level. MCF7 and MDA-MB-231 breast cancer cell lines were treated with EtOH 2 % (NT) or with LA (1 or 2 mM) for 48 and 72 hrs. IGF-1R mRNA level was assessed by real-time quantitative reverse transcription PCR in **A**: MCF7 (ERα+) and **B**: MDA-MB-231 (ERα-) cell lines treated with LA (1 or 2 mM). Data are normalized against 28S mRNA level used as an endogenous control. Results are expressed relative to the levels in control cells set at one. This analysis was performed 3 times.

Figure 5: LA promotes the pro-IR accumulation and reduces the mature IR-α expression. Pro-IR and mature IR-α expressions were detected by Western blot analysis after 48 and 72 hrs of Ethanol (EtOH 2 %) treatment or LA treatment (1 or 2 mM) in **A**: MCF7 (ERα+) and **B**: MDA-MB-231 (ERα-) cell lines.

Figure 6: LA inhibits the Notch3 maturation in breast cancer cell lines. The mature Notch3 protein expression was detected by Western blot analysis after 48 hrs of Ethanol treatment (EtOH 2 %) or LA treatment (2 mM) in **A**: MCF7 (ERα+) and **B**: MDA-MB-231 (ERα-) cell lines.

Figure 7: LA has a reversible effect on furin downregulation. The furin mRNA level is re-expressed after 24 hrs in LA-free medium. furin mRNA level was assessed by real-time quantitative RT-PCR in **A**: MCF7 (ERα+) and **B**: MDA-MB-231 (ERα-) cell lines treated with LA (1 or 2 mM) for 36 or 48 hrs followed by 24 hrs of incubation with LA-free medium. Data are normalized against 28S mRNA level used as an endogenous control. Results are expressed relative to the level in control cells set at one. This analysis was performed 3 times.

Figure 1


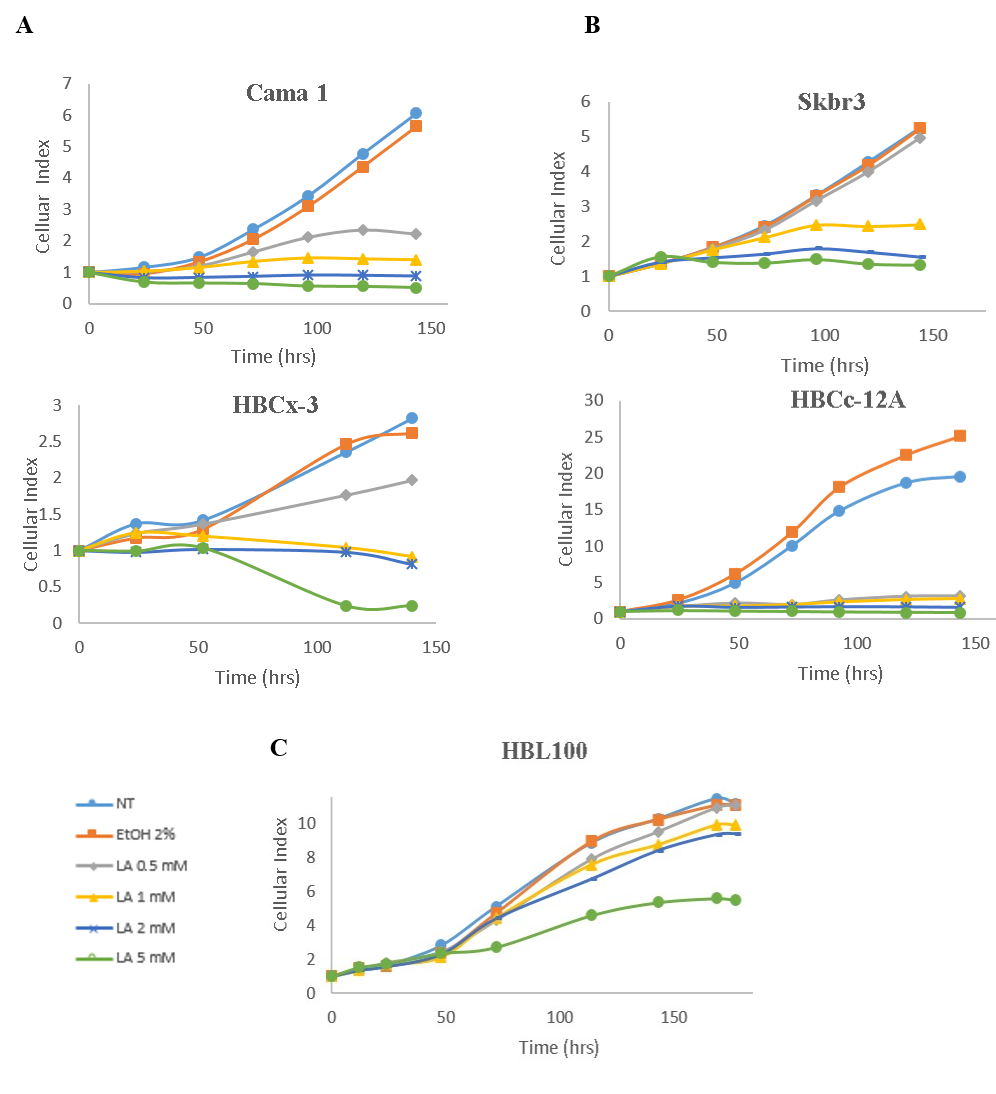


Figure 2


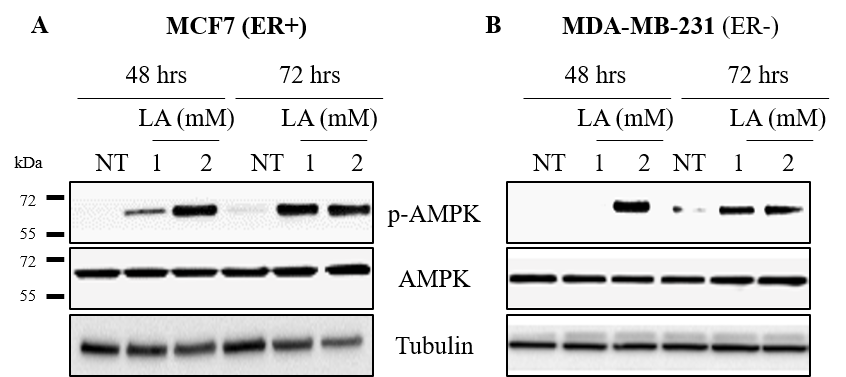


Figure 3


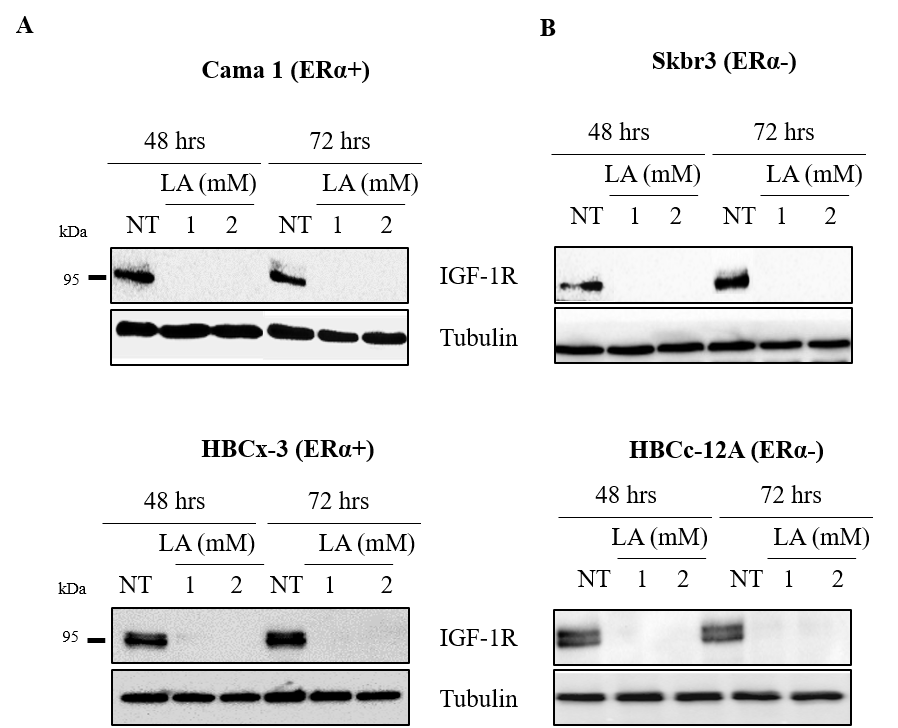


Figure 4


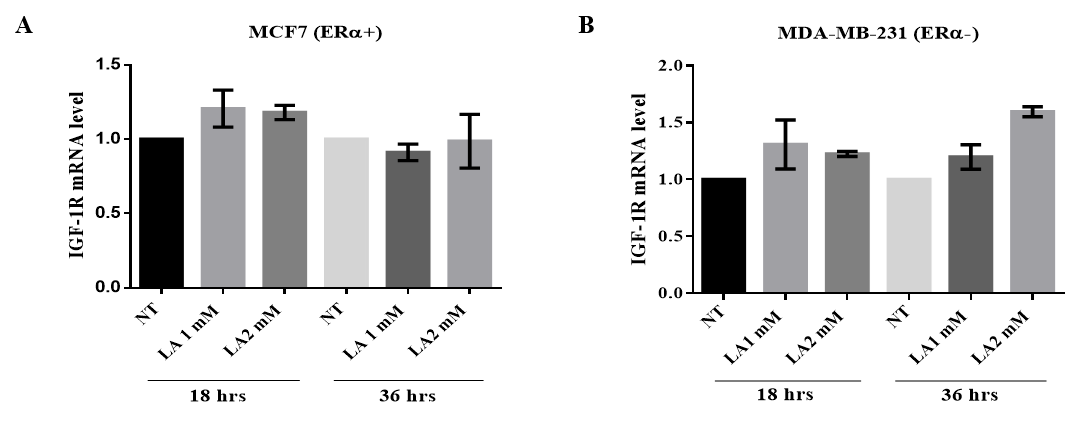


Figure 5


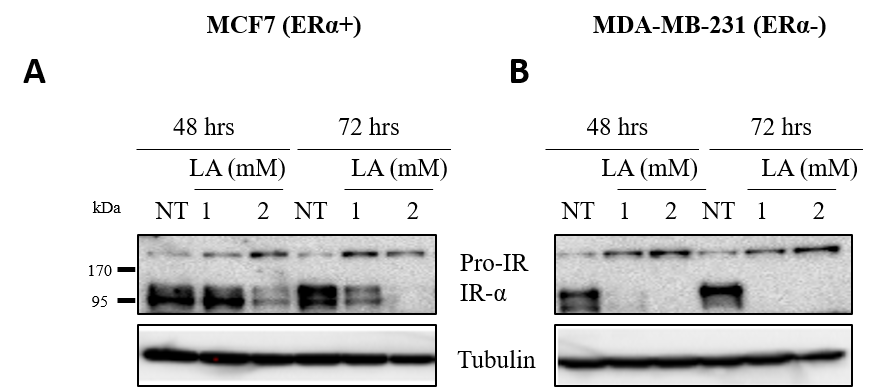


Figure 6


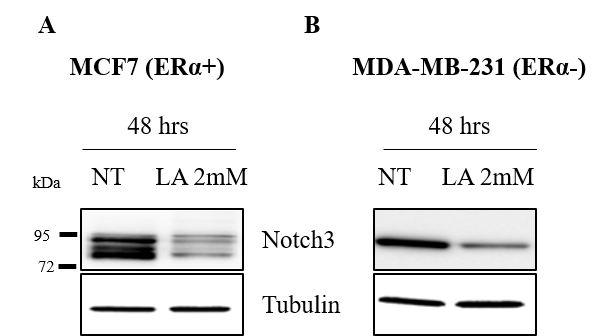


Figure 7


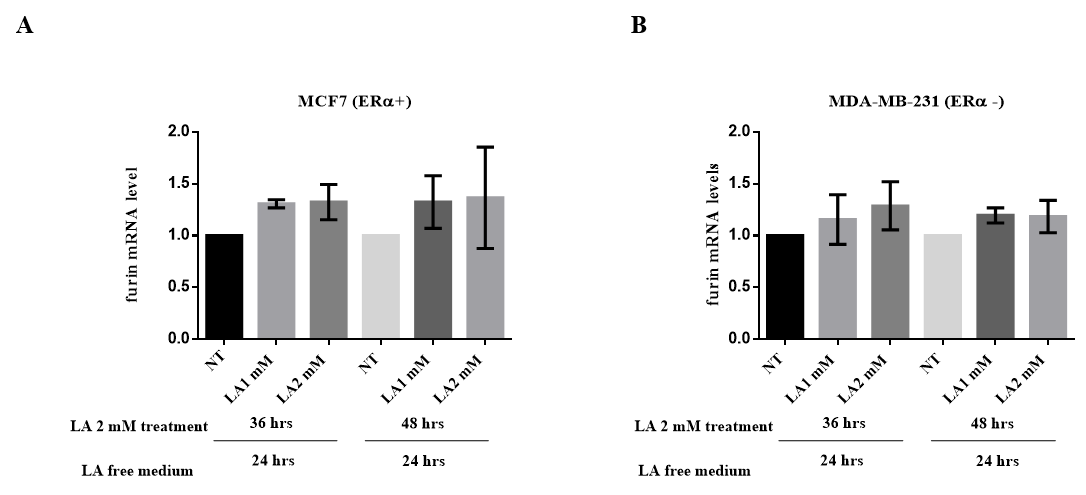

Supplement: Supplementary file 1 — supplementary data [file 41416_2020_729_MOESM1_ESM.doc]
